# Actualización de la distribución espaciotemporal de *Aedes albopictus* en Ecuador

**DOI:** 10.7705/biomedica.7719

**Published:** 2025-08-11

**Authors:** Patricio Mora, Paúl Quinatoa, Diego Morales

**Affiliations:** 1 Centro de Referencia Nacional de Vectores, Instituto Nacional de Investigación en Salud Pública - INSPI, Quito, Ecuador Instituto Nacional de Investigación en Salud Pública - INSPI Instituto Nacional de Investigación en Salud Pública - INSPI Quito Ecuador

**Keywords:** Aedes, epidemiología, arbovirus, entomología, Ecuador., Aedes, epidemiology, arboviruses, entomology, Ecuador.

## Abstract

**Introducción.:**

*Aedes albopictus* se ha expandido a 85 países por su capacidad de adaptación a las nuevas condiciones climáticas. En Ecuador, fue identificado por primera vez en Guayaquil en el 2017 y se ha distribuido a nuevas áreas geográficas. Su importancia para la salud pública se ha relacionado con su capacidad vectorial para la transmisión de arbovirus.

**Objetivo.:**

Caracterizar los criaderos de *Ae. albopictus* que han favorecido su dispersión hacia localidades geográficas de Ecuador sin reportes previos.

**Materiales y métodos.:**

Entre el 2018 y el 2024, se hicieron muéstreos entomológicos en 18 provincias y se recolectaron larvas en diversos tipos de criaderos. Se elaboraron mapas de la distribución espaciotemporal de *Ae. albopictus* y se analizaron las diferencias significativas en el número de mosquitos entre los diferentes criaderos.

**Resultados.:**

Entre el 2018 y el 2024, la presencia de *Ae. albopictus* se registró en 311 localidades de las provincias de Manabí, Guayas, Santo Domingo de los Tsáchilas, Orellana, Imbabura y Sucumbíos. Se identificaron latas, llantas y tanques plásticos, como criaderos efectivos para su crecimiento, además de contenedores naturales, como entrenudos de bambú, charcos y axilas de bromelias, aunque con menor frecuencia. La correlación de Spearman mostró una relación positiva y estadísticamente significativa (p < 5,0 x 10^-4^) entre la frecuencia de criaderos y el número de individuos.

**Conclusiones.:**

Las actividades de vigilancia entomológica ayudaron a documentar la distribución de *Ae. albopictus* en las áreas urbanas de seis provincias de Ecuador. Se identificó una correlación positiva entre el número de mosquitos y la frecuencia de criaderos encontrados en llantas, depósitos diversos y tanques plásticos.

## Abstract (en)

**Introduction.:**

*Aedes albopictus* has spread to 85 countries due to its ability to adapt to new climatic conditions. In Ecuador, it was first identified in Guayaquil in 2017 and since then it has expanded into new geographical areas. Its importance for public health has been linked to its vector capacity to transmit arboviruses.

**Objective.:**

To characterize *Ae. albopictus* breeding sites that have contributed to its dispersal into geographical regions of Ecuador without prior records.

**Materials and methods.:**

Entomological sampling was conducted in 18 provinces between 2018 and 2024. We collected larvae from various types of water-holding containers. We generated spatiotemporal distribution maps of *Ae. albopictus* and performed statistical analyses to identify significant differences in the number of mosquitoes between different breeding sites.

**Results.:**

Between 2018 and 2024, the presence of *Ae. albopictus* was recorded in 311 localities in the provinces of Manabí, Guayas, Santo Domingo de los Tsáchilas, Orellana, Imbabura, and Sucumbíos. Cans, tires, and plastic tanks were identified as effective breeding sites, while natural containers, such as bamboo internodes, puddles, and bromelia axils, were less frequent. Spearman’s correlation showed a significant positive relationship (p value < 5.0 × 10^-4^) between the frequency of breeding sites and the number of individuals.

**Conclusions.:**

Entomological surveillance activities contributed to establish *Ae. albopictus* distribution in urban areas of six provinces in Ecuador. A positive correlation was identified between the number of mosquitoes and the frequency of breeding sites, such as tires, tanks, and other water-holding plastic containers.

El mosquito tigre asiático, *Aedes albopictus* (Skuse, 1894), es originario de las regiones tropicales y subtropicales, y ha logrado extenderse a nivel mundial, excepto en la Antártida. Su gran capacidad de adaptación a ambientes con temperaturas invernales suaves (0^o^ a -5^o^ C), y a periodos largos de sequía y mucha humedad, le ha permitido desplazarse a diferentes regiones como Siberia y el norte de China ^1^^,^^2^.

Al igual que *Aedes aegypti*, esta especie puede desarrollarse en ambientes peridomésticos, donde se alimenta de la sangre de humanos y animales, y en los cuales puede poner sus huevos en una variedad de contenedores de agua naturales y artificiales ^3^. La transmisión de arbovirus por *Ae. albopictus* se ha vinculado con los brotes epidémicos de fiebre de chikunguña (CHIKV), dengue (DENV) y enfermedad por el virus del Zika (ZIKV) en Gabón y África Central; y de CHIKV y DENV en Italia y Francia ^4^^,^^5^.

En condiciones de laboratorio, *Ae. albopictus* ha sido incriminado en infecciones ocasionadas por 32 arbovirus. Sin embargo, su capacidad para transmitir cualquiera de ellos no se ha demostrado con claridad ^6^. Actualmente, *Ae. albopictus* se ha reportado en 85 países, con una distribución global en latitudes más altas en el hemisferio norte (Europa, Estados Unidos y Japón), además de regiones tropicales y subtropicales.

En la región de las Américas, su introducción se ha relacionado con la comercialización internacional de neumáticos, plantas de interior y tocones de bambú, entre otros productos que provienen de los países asiáticos. Hasta la fecha, se ha reportado la distribución de este mosquito en 21 de los 44 países de América, excepto en Chile y Perú, donde aún no se ha confirmado la presencia de esta especie ^7^^,^^8^.

A nivel local, la dispersión de *Ae. albopictus* puede asociarse con las redes viales nacionales e internacionales que facilitan su colonización entre los países de América ^9^^-^^11^. Factores climáticos como la temperatura y la precipitación han favorecido la dispersión de esta especie ya que pueden prolongar o acortar significativamente la duración de los estadios larvales, al generar nuevos ambientes acuáticos para su desarrollo. Los factores socioeconómicos también han demostrado tener una influencia representativa mediante el uso o cobertura del suelo, el tipo de urbanización y la densidad de las poblaciones humanas, pues pueden impulsar la distribución y densidad poblacional de *Ae. albopictus*^12^^,^^13^.

En Ecuador, *Ae. albopictus* fue reportado por primera vez en el 2017 en Guayaquil, en un área urbana con alta densidad poblacional ^14^. Esta ciudad se encuentra en la costa del Pacifico y cuenta con uno de los puertos principales del país donde llega más del 80 % de las importaciones comerciales ^14^. En el 2022, se reportó la presencia de esta especie en las localidades de Lita, Imbabura y en Puerto Francisco de Orellana.

Mediante los estudios filogenéticos, se demostró que los individuos de *Ae. albopictus* registrados en el país tienen dos haplotipos: el haplotipo 1 (H1) se encuentra en Guayaquil y Puerto Francisco de Orellana en Ecuador, y está presente en la región de las Américas, Europa, África y Asia; el haplotipo 2 (H2), encontrado en la localidad de Lita, estaría geográficamente restringido a países ecuatoriales tropicales de África (Camerún, Congo) y Asia (Singapur) ^15^.

El cambio climático y el desarrollo urbanístico están contribuyendo a la expansión del hábitat ideal de *Ae. albopictus*, lo que le ha permitido adaptarse a mayores altitudes y latitudes ^16^. El comportamiento alimentario antropofílico y su proliferación en contenedores domésticos han llevado a que *Ae. albopictus* comparta criaderos con *Ae. aegypti*, así como los mismos recursos alimenticios requeridos para su desarrollo ^17^. La relación de *Ae. albopictus* como potencial vector de más de 20 arbovirus -entre ellos dengue, chikunguña y Zika- resalta la necesidad de evaluar, con precisión y en el tiempo, el riesgo probable de transmisión para facilitar la toma de decisiones en las intervenciones de salud pública ^16^.

Las actividades de vigilancia entomológica han permitido caracterizar el riesgo de enfermedades transmitidas por vectores, ya que proporcionan información para el diseño de las estrategias de control. Sin embargo, la distribución de *Ae. albopictus* en Ecuador no se ha establecido con exactitud desde sus primeros registros. El comprender el alcance territorial de esta especie y su competitividad para la transmisión de arbovirus, resulta fundamental para la implementación de medidas eficaces de vigilancia entomológica.

El objetivo de este estudio fue caracterizar los criaderos de mosquitos que han favorecido la dispersión de *Ae. albopictus* y presentar los nuevos registros geográficos de su distribución en Ecuador. Con esta información se pretende establecer áreas centinelas para la vigilancia entomológica, en función de los factores sociales y climáticos que pueden promover su crecimiento poblacional.

## Materiales y métodos

### Área de estudio

Ecuador se compone de cuatro regiones geográficas: la costera, la amazónica, la sierra y la de transición entre la costa y la Amazonia. La región costera, que se extiende hasta los 1.000 metros sobre el nivel del mar (msnm) de los Andes, tiene temperaturas de 20,2 a 25,7 °C y es la de mayor incidencia de arbovirosis. La región amazónica presenta un clima tropical muy húmedo, con temperaturas promedio de 24 a 25 °C. La región sierra alcanza hasta los 4.000 msnm y presenta una amplia variabilidad climática. La región de transición entre la costa y la Amazonia presenta climas templados semihúmedos y tropicales húmedos, y un gradiente térmico de aproximadamente 5 °C por cada 1.000 m de elevación ^18^.

Las labores de vigilancia se llevaron a cabo en diferentes zonas geográficas del país, principalmente en las provincias con regiones ecológicas por debajo de los 2.000 msnm, de clima cálido tropical o subtropical y con registros previos de *Ae. albopictus*. El comportamiento ecológico registrado del mosquito permitió identificar localidades con gran flujo comercial, ciudades con puertos marítimos o aéreos y otras áreas geográficas que presentaban factores biológicos favorables para su desarrollo. Los análisis se hicieron al nivel administrativo de parroquia -la división política más elemental- que concentra barrios o localidades según el área urbana o rural.

### Muéstreos entomológicos

Se hicieron muéstreos en 18 provincias de Ecuador desde el 2018 hasta el primer semestre del 2024. Se seleccionaron sitios de muestreo con condiciones ecológicas, climáticas y sociodemográficas asociadas con una gran probabilidad de desarrollo del mosquito, y que formaban parte de las actividades rutinarias de vigilancia entomológica.

Los estadios inmaduros de los mosquitos fueron recolectados en localidades urbanas y rurales, de acuerdo con la programación de actividades de vigilancia entomológica de la Red Nacional de Laboratorios de Entomología del Ministerio de Salud Pública de Ecuador y el Centro de Referencia Nacional de Vectores. A partir de los nuevos registros de *Ae. albopictus* obtenidos durante los años de monitoreo, se modificaron las localidades de vigilancia entomológica para ampliar el rango geográfico de la vigilancia epidemiológica.

En cada localidad, se seleccionaron aleatoriamente alrededor de 50 viviendas para la captura manual de estadios larvales. En cada vivienda, se inspeccionaron los depósitos de agua para registrar el tipo de contenedor y determinar la presencia de larvas. Los hábitats larvales se clasificaron en contenedores artificiales, como baldes plásticos, depósitos diversos (latas, tarrinas, bebederos de animales), floreros, lavaderos, llantas, ollas, piscinas, tanques y tinas; y contenedores naturales, como entrenudos de bambú, charcos y axilas de bromellas ^19^.

Se usaron pipetas plásticas de 30 mi para recolectar la mayor cantidad de larvas por depósito de agua. Los individuos recolectados fueron transportados al insectario del Centro de Referencia Nacional de Vectores. Los estadios larvales se mantuvieron en condiciones de laboratorio a una temperatura de 26 ± 2 °C y una humedad de 60 ± 5 %, hasta alcanzar el estadio adulto. Los individuos se identificaron morfológicamente mediante claves dicotómicas de mosquitos (Díptera: Culicidae) asociados con la transmisión del virus del dengue ^20^.

### Análisis de la información

Se elaboró un formulario para recolectar los datos entomológicos de los criaderos de mosquitos inspeccionados. Se registraron las coordenadas de los lugares de recolección, las características del tipo de criadero identificado y el método de recolección utilizado. Toda la información se Ingresó en una base de datos que fue organizada según el año de recolección, el nombre de la localidad asociado con los niveles administrativos del país y el número de mosquitos identificados por contenedor.

Se elaboraron mapas de distribución espaciotemporal a partir de los registros obtenidos de *Ae. albopictus* por cada año de vigilancia, asociando la presencia o ausencia a nivel de parroquia. Una vez detectada la presencia de *Ae. albopictus* en una parroquia, se asumió su continuidad en los años siguientes. Todos los registros de la especie se representaron en un mapa a nivel nacional, para observar el establecimiento de las poblaciones. Los mapas se generaron utilizando el *software* QGIS, versión 3.24 (https://qgis.org/pt_BR/sitio/).

Se evaluó la existencia de diferencias significativas en la cantidad de mosquitos presentes en los diferentes tipos de criaderos. Para ello, se aplicó la prueba de normalidad de Shapiro-Wilk para evaluar si los datos de esta variable (N = 1.999) seguían una distribución normal. Posteriormente, se utilizó la correlación de Spearman para analizar la relación entre la frecuencia de los criaderos y el número de individuos encontrados. Se calculó el coeficiente de correlación (r) y se evaluó la significancia estadística, considerando un alfa de 0,05, para investigar el vínculo entre cada variable y grupo, según sus niveles de similitud. Los datos y gráficos generados se elaboraron con RStudio, versión 2023.06.2 (Build 561, http://www.rstudio.com/).

## Resultados

**Figura 1.**
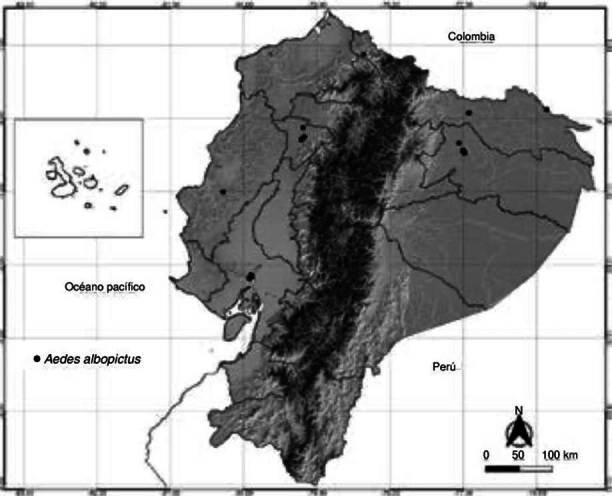
Registros de circulación de poblaciones de *Aedes albopictus* en Ecuador

Se registró la presencia de *Ae. albopictu* en 311 localidades monitoreadas desde el 2018 a nivel nacional (figura 1). En la región costera, la zona de dispersión de *Ae. albopictus* se encuentra entre las provincias de Manabí y Guayas, desde los 300 hasta los 860 msnm; y, para la provincia de Santo Domingo de los Tsáchilas, a los 1.005 msnm. En la región amazónica, la dispersión ha alcanzado las áreas urbanas de las provincias de Orellana y Sucumbíos, que se encuentran por debajo de los 870 msnm. En la región de los Andes, el único registro se encontró en la provincia de Imbabura, en áreas de transición con clima subtropical a una altura de 551 msnm.

**Figura 2.**
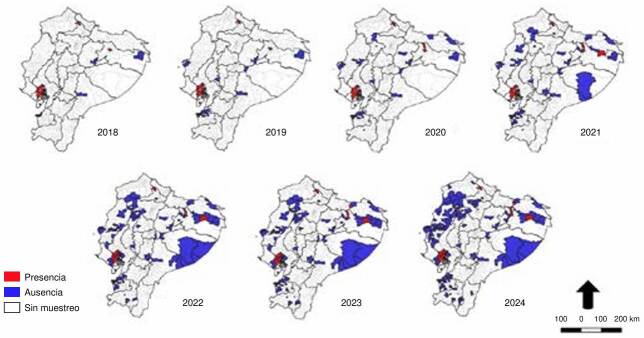
Registro de distribución espacial y temporal de *Aedes albopictus* por parroquia desde el 2018 al 2024

Según el análisis de la distribución de *Ae. albopictus* por parroquia en el 2018, esta especie se encontraba en dos áreas geográficas: en la región amazónica, en ambientes urbanos, y en la región de los Andes, en zonas rurales con clima subtropical y baja concentración de viviendas. Entre el 2019 y el 2021, los sitios de vigilancia epidemiológica se ampliaron hacia otras parroquias cercanas a los primeros lugares de identificación en la regiones costera y amazónica. Como resultado, se obtuvo un nuevo registro de la especie en parroquias de la región oriental. En el 2022, se registró la presencia de *Ae. albopictus* en una parroquia de la provincia de Manabí y, hasta el 2024, se ha reportado su presencia en nuevas parroquias de la región amazónica, como en la provincia de Sucumbíos -fronteriza con Colombia- y en la zona de transición entre la costa y la región andina, en la provincia de Santo Domingo de los Tsáchilas (figura 2). Los sitios de vigilancia entomológica se han incrementado en las regiones costera y amazónica, manteniendo puntos de interés en las áreas de transición ecológica y ecosistémica que proporcionan condiciones idóneas para el desarrollo del mosquito.

**Figura 3.**
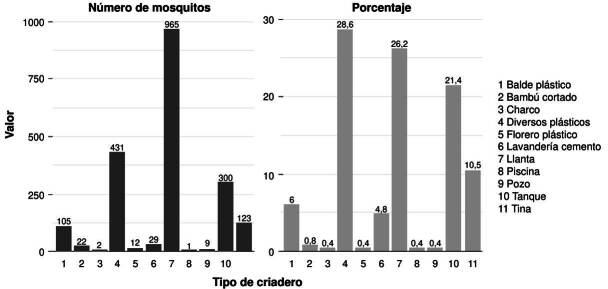
Izquierda: número de individuos recolectados de *Aedes albopictus* por tipo de criadero; derecha: porcentajes de abundancia relativa del mosquito

Se identificaron 248 criaderos de mosquitos con la presencia de estadios larvales de *Ae. albopictus*. Los hábitats más frecuentes fueron los contenedores artificiales, como los depósitos diversos (latas, tarrinas, bebederos de animales: 28,6 %), las llantas (26,2 %) y los tanques plásticos (21,4 %); y en menor medida, los contenedores naturales, como bambú cortado y charcos. La frecuencia de criaderos naturales con *Ae. albopictus* fue menor de 1 % (figura 3).

Se identificaron 1.999 individuos de *Ae. albopictus*, encontrados con mayor frecuencia en los criaderos artificiales, como llantas (n = 965), diversos plásticos (n = 431) y tanques plásticos (n = 300). En los otros contenedores de origen natural, como bambú cortado (n = 22), charco (n = 2) y piscina (n = 1), se registró una menor cantidad de individuos. En cada contenedor, el número de especímenes recolectados fue independiente de la frecuencia de contenedores observados.

La correlación de Spearman (r = 0,9677) evidenció una relación positiva entre la frecuencia de los criaderos de mosquitos y el número de individuos encontrados. Se determinó que los criaderos más frecuentes (depósitos diversos, llantas y tanques) están asociados con el desarrollo de *Ae. albopictus* (p < 5,0 x 10^-4^).

## Discusión

Los registros de *Ae. albopictus*, desde el 2018 hasta el 2024, muestran que el mosquito se ha desplazado desde Guayaquil (lugar del primer registro) a otras provincias de las regiones costera y amazónica. En estas áreas geográficas predomina un clima tropical de árido-seco a húmedo en la región costera y, un clima tropical muy húmedo, en la región amazónica. La capacidad de adaptación de *Ae. albopictus* le facilita desarrollarse en zonas geográficas con climas templados y tropicales, con rangos de temperatura bajo los -2 °C, con un óptimo de 29,7 °C ^21^^-^^23^. La adaptabilidad ecológica y la capacidad de los huevos de esta especie para sobrevivir a temperaturas tan bajas han permitido su rápida dispersión en latitudes con climas templados y estacionales, fuera de su rango de distribución nativo ^24^. En Colombia, se ha evidenciado que la distribución de esta especie comprende altitudes que van desde los 0 a los 1.800 msnm. Los modelos de nicho ecológico demuestran que posee una gran capacidad de adaptación en una parte significativa de la Amazonia ^25^.

Otros registros de esta especie, en la región amazónica de Brasil, determinaron que los períodos con mayores precipitaciones favorecían la aparición de nuevos sitios de reproducción -adecuados para la oviposición y el desarrollo de formas inmaduras-, lo que llevó a un aumento de la población de *Ae. albopictus*^26^. Este factor puede ser determinante en la generación de nuevos criaderos en las zonas urbanas y en la expansión de esta especie en localidades de la región amazónica de Ecuador que presentan condiciones similares. En zonas de transición -forestales a urbanas-, *Ae. albopictus* fue más abundante en áreas con cobertura vegetal de densidad alta o intermedia ^27^.

Desde el 2018, las actividades de vigilancia entomológica han identificado el desplazamiento de *Ae. albopictus* a la provincia de Orellana y, posteriormente, en el 2023, a la provincia de Sucumbíos, en parroquias de la región amazónica fronterizas con Perú y Colombia. Estas áreas geográficas están influenciadas por actividades de extracción petrolera, comercio, agricultura y, en menor intensidad, minería a pequeña escala, turismo y manufactura ^28^. En el caso de la provincia de Orellana, las identificaciones se hicieron en una localidad con uno de los principales puertos de la región amazónica; esta región es fronteriza entre Ecuador y Perú a lo largo del afluente del río Ñapo, que desemboca en el río Amazonas en el departamento de Loreto.

En la mayoría de los países de las Américas, la introducción de *Ae. albopictus* se ha asociado con el comercio intercontinental de neumáticos usados y otras mercancías que transportan huevos de este mosquito ^29^. Los puertos marítimos se han considerado como vías primarias para el ingreso de especies exóticas a zonas geográficas donde naturalmente no podrían llegar, como es el caso de *Ae. albopictus*. La ubicación estratégica de los puertos fluviales y marítimos, junto con las principales rutas de transporte terrestre que conectan diferentes regiones del país, pueden proporcionar una vía para la entrada y salida de vectores a nuevas áreas geográficas ^30^^,^^31^. En un estudio mediante modelos matemáticos, se determinó que el transporte pasivo mediado por los camiones de carga desempeña un papel importante en la dispersión de *Ae. aegypti* y en su capacidad potencial para transmitir arbovirus a escala nacional en Colombia ^32^.

En la región amazónica de Ecuador, el comercio de insumos para extracción petrolera, minería y agricultura, han facilitado la dispersión de *Ae. albopictus* en las zonas central y norte, con la posibilidad de extenderse hacia el sur de la Amazonia. El puerto de Orellana es otra puerta de entrada para esta especie, al ser una fuente fluvial primaria que conecta con la región amazónica de Brasil, donde continuamente se realizan actividades comerciales para abastecer de productos a las comunidades asentadas a lo largo del río Napo.

Los criaderos con mayor proporción de *Ae. albopictus* fueron los depósitos plásticos diversos, seguidos por las llantas y los tanques plásticos. En conjunto, estos contenedores artificiales contribuyeron con el 98,8 % de los especímenes recolectados, en comparación con el 1,2 % encontrado en los criaderos naturales. Estos contenedores artificiales generalmente se encuentran en el exterior de la vivienda, expuestos a condiciones climáticas que conservan la humedad (evitando la evaporación del agua) y favorecen la reproducción de mosquitos.

En estudios anteriores, se había sugerido que *Ae. albopictus* se encontraba exclusivamente en hábitats naturales, como huecos de árboles, cortes de bambú y bromelias; sin embargo, los reportes de España y Brasil han evidenciado su capacidad adaptativa en las áreas urbanas mediante la colonización de diferentes sitios de reproducción artificial, como bebederos de animales, tarros de agua, llantas y tanques de cemento ^26^^,^^33^.

Estos datos sugieren que las poblaciones de mosquitos se han adaptado a los entornos antropogénicos y son capaces de utilizar diversos tipos de criaderos, incluidos numerosos contenedores generados por el hombre.

En Ecuador, los contenedores de agua, como llantas, tanques plásticos y de metal, lavaderos, tarrinas, bebederos de animales, entre otros, se encuentran alrededor de las viviendas y permiten el desarrollo de *Ae. aegypti* y *Ae. albopictus*. En las regiones costera y amazónica, la disposición de estos contenedores es permanente y ha facilitado la dispersión de poblaciones de mosquitos, en conjunto con el comercio y la migración humana. Los estudios de otras poblaciones de *Ae. albopictus* determinaron que esta especie prefiere los ambientes peridomiciliarios, donde coexiste con *Ae. aegypti*, lo que indicaría una competencia interespecífica por alimento y refugio que, a su vez, incrementa su potencial epidemiológico ^34^^,^^35^. La presencia de *Aedes albopictus* en estos entornos tiene implicaciones ecológicas y epidemiológicas relevantes, ya que evidencia la adaptación de esta especie a los ambientes antropogénicos y su potencial para transmitir diversos arbovirus de importancia en salud pública ^36^.

Los análisis determinaron una correlación positiva entre la cantidad de individuos de *Ae. albopictus* y la frecuencia de los criaderos. Aunque los contenedores plásticos diversos fueron los más frecuentes en el muestreo, la cantidad de individuos hallados en ellos fue baja en comparación con otros depósitos inspeccionados. Los contenedores con mayor cantidad de *Ae. albopictus* fueron las llantas y los tanques plásticos, con un registro máximo de 965 individuos en las llantas. Estos resultados sugieren que, en el país, las poblaciones *Ae. albopictus* se encuentran asociadas con este tipo de contenedor, lo que ha facilitado su permanencia y dispersión hacia diferentes localidades.

La adaptabilidad de *Ae. albopictus* a las llantas se debe a la generación de microhábitats, ya que este tipo de material proporciona aislamiento y condiciones isotérmicas adecuadas, especialmente en climas fríos, lo que permite su supervivencia durante el invierno, según lo reportado en poblaciones de Italia ^37^.

En el caso de los depósitos diversos, se ha demostrado ampliamente que los recipientes no degradables son los sitios de reproducción más frecuentes, ya que el número de larvas aumenta con la disponibilidad de los contenedores de desechos. Al parecer, el material plástico favorece la formación de diversos tipos de hábitats ideales para la reproducción de *Ae. albopictus* y *Ae. aegypti*^38^. Estos factores han permitido la supervivencia y la expansión de *Ae. albopictus* a otras áreas geográficas de Ecuador, con la posibilidad de que alcance zonas de mayor altitud, donde las condiciones medioambientales podrían ser favorables para su establecimiento, como las estribaciones de la cordillera de los Andes con climas subtropicales.

Las actividades de vigilancia entomológica de este estudio tuvieron lugar en áreas urbanas, periurbanas y rurales. Sin embargo, el registro de presencia de *Ae. albopictus* fue predominante en las áreas urbanas con gran densidad poblacional, lo que indicaría una asociación de esta especie con los ambientes completamente urbanizados. Las carreteras son un factor determinante para la distribución de *Ae. albopictus* y su dispersión se podría vincular al comercio de llantas, ya que es el criadero más favorable para su dispersión.

En los muéstreos realizados, la ciudad de Santo Domingo registró la población más numerosa de *Ae. albopictus*. Esta localidad se caracteriza por unir las principales rutas comerciales por vía terrestre, ya que conecta los puertos marítimos con localidades de las regiones andina y amazónica. En Panamá, en estudios de modelos matemáticos, se determinó que las carreteras pueden usarse como un factor independiente para predecir la expansión futura de *Ae. albopictus*^9^. Este hallazgo contrasta con los reportes europeos, que señalan que el transporte pasivo de larvas se produce en recipientes abiertos que acumulan agua, como los neumáticos usados, mientras que los adultos pueden ser transportados dentro de la cabina de automóviles y camiones.

En este estudio no se consideró el análisis de correlación entre variables que favorecen la dispersión de *Ae. albopictus* mediante modelos predictivos. Sin embargo, la red vial del país y las actividades humanas ofrecen la posibilidad de identificar otras áreas idóneas para su establecimiento, en las cuales sería necesario intensificar las actividades de vigilancia entomológica ^9^^,^^39^.

En el presente estudio, se presenta una actualización de los patrones de distribución de *Ae. albopictus* en Ecuador. Se documenta su expansión desde Guayaquil (2017) hacia dos provincias de la región costera, dos de la región amazónica y una de la región interandina, en una zona de transición climática. Después de siete años de actividades de vigilancia entomológica, se ha identificado un avance cronológico de esta especie hasta la región amazónica, posiblemente influenciado por las actividades petroleras y comerciales predominantes en la zona, así como por otros factores antropogénicos que han favorecido su establecimiento.

Las llantas se identificaron como uno de los criaderos principales para el desarrollo de *Ae. albopictus* que han facilitado su dispersión a diferentes localidades. La alta densidad poblacional, la movilidad humana y la presencia de criaderos potenciales en los ambientes domiciliarios, deben incluirse en la vigilancia entomológica para identificar otras áreas geográficas donde aún no se ha registrado la presencia de esta especie.

Además, debido a la creciente evidencia que respalda la gran capacidad de *Ae. albopictus* para adaptarse a nuevas regiones y su potencial para la transmisión horizontal y vertical del DENV en ambientes urbanos y periurbanos, es necesario fortalecer la vigilancia entomovirológica a nivel nacional.
